# Toward Reliable and Reproducible Research in Organic Photocatalysis by Carbon Nitride

**DOI:** 10.1021/acscatal.5c04794

**Published:** 2025-09-19

**Authors:** Gaia Grando, Giuseppe Sportelli, Gaia Castellani, Giacomo Filippini, Maurizio Prato, Michele Melchionna, Paolo Fornasiero

**Affiliations:** a Department of Chemical and Pharmaceutical Sciences, University of Trieste, and INSTM Trieste Research Unit, via Licio Giorgieri 1, Trieste 34127, Italy; b Centre for Cooperative Research in Biomaterials (CIC BiomaGUNE), Basque Research and Technology Alliance (BRTA), Paseo de Miramón 194, Donostia San Sebastián 20014, Spain; c Basque Fdn Sci, Ikerbasque, Bilbao 48013, Spain; ∥ Center for Energy, Environment and Transport “Giacomo Ciamician” and ICCOM-CNR Trieste Research Unit 9315 University of Trieste, via Licio Giorgieri 1, Trieste 34127, Italy

**Keywords:** reproducibility, carbon nitrides, organic synthesis, heterogeneous photocatalysis, slow chemistry

## Abstract

The rate of scientific publications has grown exponentially over the past few decades, but this has come at the expense of reproducibility. In this context, fields like organic photocatalysis and materials synthesis have also been affected. This Perspective aims at providing general guidelines to increase trustworthiness and favor reproducibility for those interdisciplinary researchers working on organic photocatalytic transformations catalyzed by carbon-nitride-based materials. Thus, the article focuses on the importance of accurately reporting and describing all the stages of experimental work, from the photocatalyst synthesis and characterization to the evaluation of the reaction conditions, control experiments, and—more generally—all the details that may ensure reproducibility. Additionally, we investigate and discuss the risks of falling into inadequate research practices, emphasizing that irreproducibility in science is a major problem that undermines the utility and credibility of scientific research. These aspects are crucial for the scientific community, and we emphasize the need to raise awareness and educate researchers toward best experimental practices.

## Graphical abstract

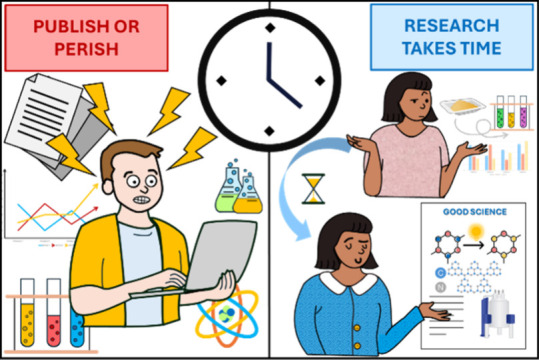

## 1 Introduction

High-quality research demands meticulous, patient and unbiased analysis of all the acquired data. Hasty hypotheses and conclusions, imposed by the frenzy of the “publish or perish” attitude, can be highly toxic for scientific developments. In this context, we wish to highlight a couple of historical examples that underscore the core values of research (Figure 1a). For instance, the development of the blue light emission diode (LED) by Prof. Shuji Nakamura required five uninterrupted years of work before making the breakthrough with the first article on the subject. Noteworthily, Prof. Nakamura proceeded to publish only after ensuring the robustness of his results, despite the pressure from his company.^1^ Similarly, it took almost a century (and several generations of scientists) before Einstein’s postulation of the existence of gravitational waves was experimentally confirmed.^2,3^ The bottom line of these two examples is that the most important scientific discoveries, namely those that make human knowledge leap forward and start revolutions, are typically long-hatching processes.

**Figure 1:**
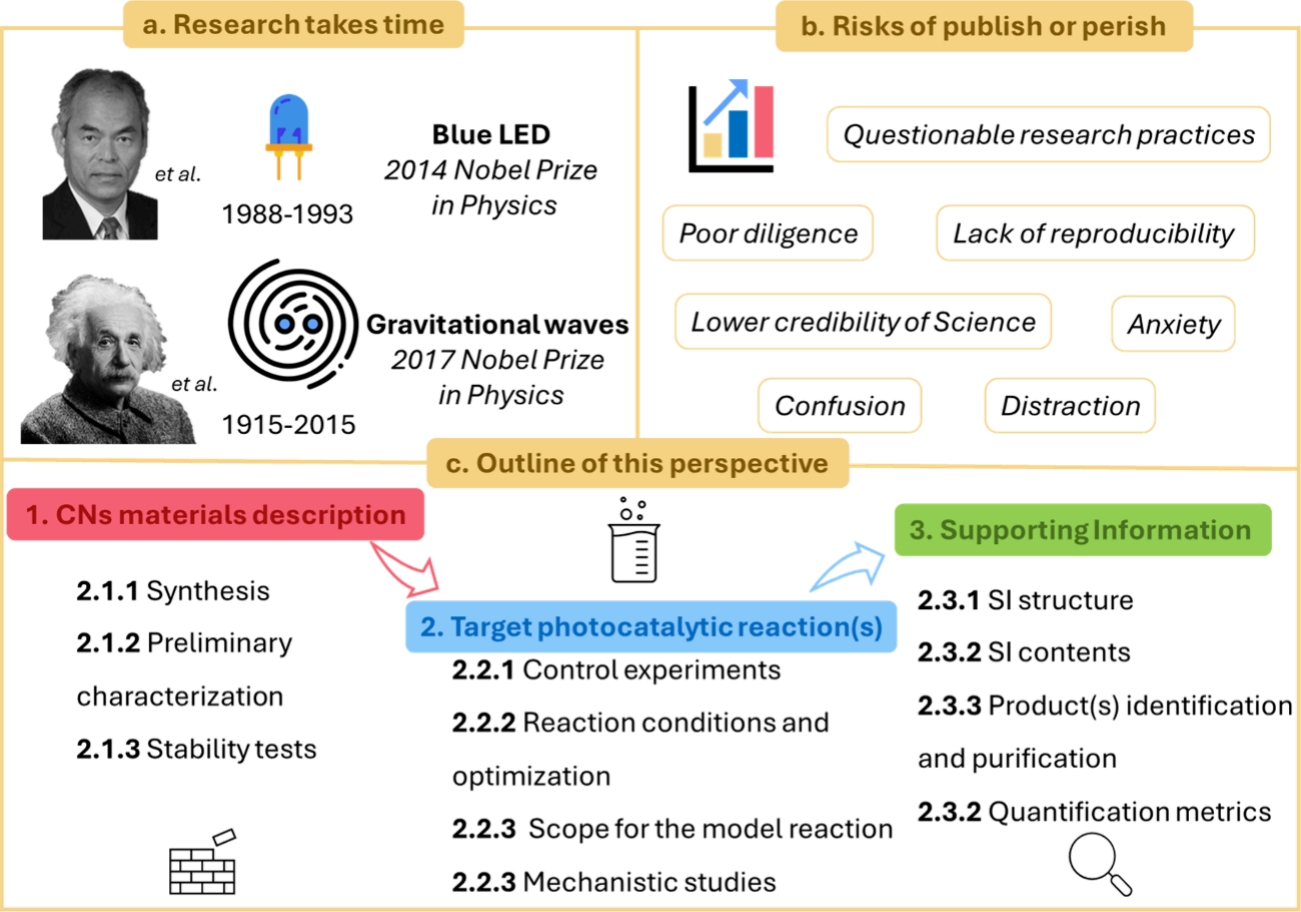
a) Notable examples of long-term research. b) Keywords related to the risks of the “publish or perish” culture. c) Scheme of the key points of a research article on organic photocatalytic transformations using CNs.

A worryingly trend in today’s research features an exponentially growing number of annually published articles, together with a growth in the number of journals to accommodate them.^4^ It is clear that the “publish or perish” culture is generating an uncontrolled avalanche effect, which diminishes the reliability of current science and somehow mortifies research (Figure 1b). Even worse, such an attitude also dictates the trends of research, establishing the wrong criterion that chances to publish in high impact journals are higher for certain topics. This is a concern closely related to the concept of “publication bias”, defined as “*the failure to publish the results of a study on the basis of the direction or strength of the study findings*”, by which scientists avoid to publish some negative or noncompetitive results to prevent overshadowing the positive aspects of their work and risking rejection.^5–8^ Conversely, science should focus on addressing specific problems or solving pending scientific issues, not on generating lists of high-impact publications to improve personal metrics.^9^ An immediate and concrete problem generated by distracted research practices is the dilemma of reproducibility of scientific results.^10^ The anxiety for fast publication often leads to poor diligence, whereby, for example, a convenient selection of the results to be reported is made, while contradictory data are somehow neglected. This makes it impossible for other groups to reproduce the same results, and it is not uncommon to encounter articles on the same topic with contrasting conclusions, thus increasing the level of confusion and uncertainty. A direct correlation has been recognized in a survey published by the journal Nature in 2016, in which 60% of respondents attributed the pressure to publish and selective reporting as one of the main causes of the reproducibility crisis.^10^ In a study published in 2017, Grimes et al.^11^ strongly criticized the current system of using the number of publications as the primary measure of academic success, since it can undermine public confidence in science.

This reproducibility crisis affects all the scientific fields, including materials science, organic synthesis and catalysis.^12^ In this context, organic photocatalytic transformations using carbon nitrides (CNs) remain a vibrant research area, so the large volume of publications on this topic in recent years could suggest susceptibility to reproducibility issues.^13^ Polymeric carbon nitride (PCN) is a semiconductor composed primarily of sp^2^-hybridized carbon and nitrogen atoms arranged in a π-conjugated planar structure. The band gap of PCN is typically around 2.7 eV, allowing visible-light absorption, which makes it a suitable for photocatalysis.^14^ PCN and its derivatives have been employed in photocatalysis in the last two decades, from the first applications in H_2_ evolution, to many other processes such as CO_2_ reduction, pollutant removal and organic synthesis.^15–18^ In all these applications several parameters must be taken into account to describe in reproducible terms the CN-catalyzed processes, focusing our attention to organic synthesis, both the catalytic systems and the reaction outputs description have their challenges. Great attention must be taken in reporting all the details of catalyst synthesis and characterization, reaction conditions, control experiments, and product characterization which are the main sensitive points that hinder reproducibility. In particular, some intrinsic irreproducibility can be brought up by the limit of detection and the sensitivity of the analytical techniques used, which reinforces the idea of comparing the same output via multiple methods.

In this Perspective, we will linger on the anatomy of a research article on organic photocatalytic transformations using CN photocatalysts. We will dissect and inspect the main parts, finally attempting to draw guidelines and best practices for reporting the results in this field (Figure 1c). Our hope is to help in establishing a common framework that will facilitate the scientific community to interpret and repeat each other’s results.

## 2 Discussion

We will now provide a critical analysis of the workflow generally adopted to write research articles in the framework of reproducibility. First of all, we will provide, the three main concepts included in the term “reproducibility”, as explained by Scott et al.:^12^ *i*) *repeatability*, namely the possibility to obtain the same result many times, *ii*) *replicability*, which is the ability to reproduce someone else’s results and *iii*) *corroboration*, that is the possibility of finding similar experiments that help to elucidate the overall chemical process, so to possibly extend the approach to other topics.

As schematized in Figure 1, the articles on CNs for photocatalytic organic transformations can be typically divided into two main sections: 1) the description of the synthesis and characterization of the material and 2) the catalytic tests, incorporating conditions optimization, control experiments, the reaction scope and the proposed mechanism (in the most ideal case, supported by *in situ* or *operando* investigations, and complemented by theoretical studies).

Finally, we will also provide some suggestions on how to structure 3) Supporting Information (SI). SI can contain a notable number of experimental details that, for length restriction reasons, cannot be included in the main text and therefore are vital for reproducibility. For these reasons it can be said that poorly prepared SI can undermine the value of the entire study.^19^

### 2.1 Material Synthesis and Characterization

Irreproducibility and unreliability in synthetic methods can originate from a large number of different causes that are not always easy to avoid. Some of them, such as the presence of impurities in the reagents as well as instruments’ misfunctions and specifications can be difficult to recognize and, consequently, could be overlooked. Another common source is the insufficient level of detail in the description of the synthetic method, which sometimes originates from assuming obvious specific knowledge. However, the lack of sufficient experimental details is always very critical in material preparation, as even the slightest change in the procedure can drastically impact the material’s properties.^20,21^ The reporting of material synthesis should therefore comply with a set of well-defined guidelines.

An innovative approach in this direction has been recently suggested by Hein, Cronin and co-workers,^22^ who proposed and implemented a standardized strategy for reporting procedures. More specifically, a universal chemical programming language, called χDL, is able to encode synthetic procedures and allow the repeating of the synthesis with automated platforms. The method is, in principle, very powerful because it allows one to obtain the same material in any other laboratory with the same equipment. However, from a practical point of view, it is not yet within the reach of most researchers, given the limited availability of costly robots in universities and research centers, hence descriptive and detailed procedure, for human-made research are still necessary.^21,22^

#### 2.1.1 Material Synthesis

In the context of polymeric carbon nitride (PCN), synthetic *bottom-up* methods are widely used because of their simplicity and facile scalability. Because of such a simplicity in their preparation, many studies on structural and compositional modifications to tune CN’s properties have been carried out.^14^ Consequently, the simple traditional preparative protocol has branched out into a large number of synthetic variations. Some of them are rather sophisticated, requiring a more complex characterization of the final structure. It is obvious that a systematic, exhaustive and generally recognized framework for the description of the material synthesis and characterization is essential.^23^ We suggest that the detailed synthetic procedure of the most relevant material (or set of materials) should be given in the “*Methods*” section in a clear and precise manner. It is surprising that such a trivial step is often overlooked. In case some synthetic details do not fit the main text, an extended version should be provided in the SI, eventually including all the other reported materials that serve for the discussion of the work.

Critical information to carefully consider for completing the “*Methods*” section is the exact specifications of all reagents used, their purity grade and suppliers, the step-by-step report of the synthetic protocols (with no assumption that the lingo used is obvious to all) and the detailed description of the experimental setup. If the protocol is based on previously published work, this should be clearly referenced. A picture, or a schematic representation of the experimental setup or procedure would be extremely useful, especially in those cases in which this is not standardized (for instance, in the main text or SI). Eventually, if relevant, a video tutorial of selected steps of the syntheses can also be attached in the SI.^21^

Referring to the case of carbon nitride, most of the structural variations are based on either changes in the synthetic conditions or on the postsynthetic modifications. In both cases, it should not be considered irrelevant to describe the synthesis of the starting pristine material (e.g., polymeric carbon nitride). In fact, the slightest changes of synthetic conditions can alter the PCN’s structure, and these alterations can propagate and amplify in the successive postsynthetic modification protocols. Details to be reported include:

- any possible pretreatment;
- the specification of name, quantity and molar ratio of precursors (if more than one is used);
- the identification of the muffle’s features;
- the calcination atmosphere (with details on the gas flow rate, in case of use of controlled atmospheres);
- the temperature ramp(s), including the conditions for cooling to room temperature (e.g.; whether cooling occurred inside or outside the furnace);
- the shape and size of the crucible (and mention how the crucible is covered, e.g. lid, a piece of aluminum foil, etc.).

Finally, all treatments such as grinding (e.g.; in mortar or with ball miller), as well as the washing and drying steps that preceded the final product need to be specified. Subsequently, any postsynthetic treatment should be described to the same level of accuracy, carrying its own load of information.^24^ Even without taking into account the formation of composites with other phases,^25–27^ there is a huge number of possible protocols for PCN modifications alone. The most common are (Figure 2):

**Figure 2:**
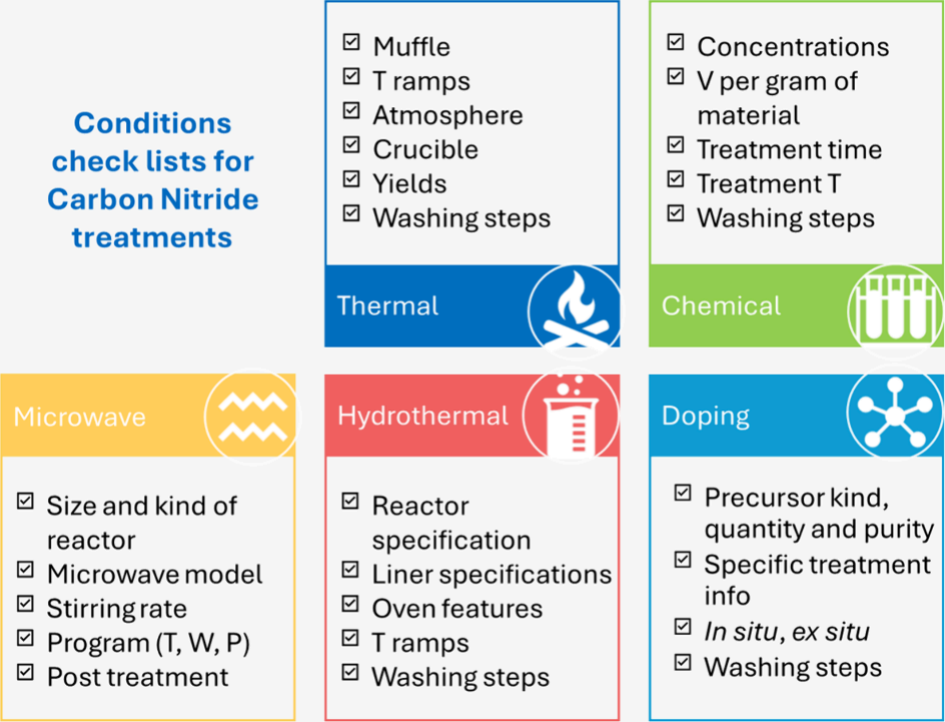
Summary of the main modifications on CNs and the relative information that should be included in the paper and/or SI. T: Temperature; V: Volume; W: Power; P: Pressure.

- thermal treatments, for which the authors should still introduce all the details already mentioned for the pristine material, like the furnace’s specifications, calcination atmosphere, and temperature ramp(s). It is also important to note that the features of the crucible remain crucial in this context, the presence or absence of a covering lid, for example, can significantly impact the properties of the final material;^28–32^

- chemical treatments, e.g. with acids or bases, which require concentration and volume of the chemical per gram of material, treatment time, and temperature must be well explained;^33,34^

- microwave treatments, which depend on the size and type of reactor, the type of microwave generator (which can span from inherently safe lab ovens to domestic ones), the stirring speed, the adopted programs (hence temperature, power and pressure regulation), and the post-treatment requirements;^35^
- hydrothermal methods, that are also used for the low-temperature synthesis of carbon nitride to tune morphology and structural features, for which the most critical points that enable reproducibility refer to reactor, liner, and oven characteristics;^36^
- metal and nonmetal doping, which is a very broad topic and difficult to generalize. For example, it should be specified if the preparation is based on in situ (i.e., concurrently with the CN synthesis) or ex-situ (i.e., as postsynthetic functionalization) methods, and thus outline all the details as above, including washing steps. In particular, in case of excess metal removed via acid leaching, the type of acid, its concentration and volume per gram of material, treatment time, and temperature should be reported.^37–39^ Other relevant details (very often not included) may possibly be added: (i) the furnace atmosphere conditioning, (ii) the quantity of material per centimeter square of crucible, (iii) the number of thermal cycles used.

Regarding *repeatability*, we note that information on this crucial topic is hardly found in scientific papers. New materials should be synthesized in replicates (i.e., more than once) with the same results within experimental uncertainty to be considered repeatable. Following the example of biology and analytical chemistry, it would be a good practice to perform at least three replicas of the material in three independent syntheses and eventually report possible observed dissimilarities, including yields.^12^ The fact that the synthesis can be successfully repeated gives important insights into the robustness of the protocol of preparation, avoiding deleterious situations in which assembly proceeds under some adventitious conditions.

The problem of the lack of replicability is widespread. For instance, a 2020 study by Agrawal et al.^40^ analyzed the replicability of 130 Metal–Organic Frameworks (MOFs). The scientists screened papers citing the original syntheses of selected MOFs to measure how frequently these materials were reported in later studies, which gives the number of successful replicas and exploitations. It turned out that it could be possible to confirm the material’s synthetic reproducibility for less than 12% of the 130 different MOFs considered, even though for 65% of the sampled materials, a modified protocol had been reported. Moreover, only 6% of these materials have been reproduced by different groups. These observations can be interpreted in light of the fact that journals typically do not publish findings without novel contributions incidentally lowering the chances to verify the reproducibility of the material. Perhaps some mentioning of negative results could be in some cases quite helpful. The low rate of reproducibility in different groups can be also since repeatability does not ensure replicability (Figure 3). We note that reproducing the same results in other laboratories and by other researchers implies the use of different machinery and instruments, variable environmental conditions, diverse synthetic skills and technical *modus operandi*. Therefore, it emerges that the process of ensuring reproducibility takes time and effort, and it is not a simple task, although, in our view, would deserve more attention.^12^

**Figure 3:**
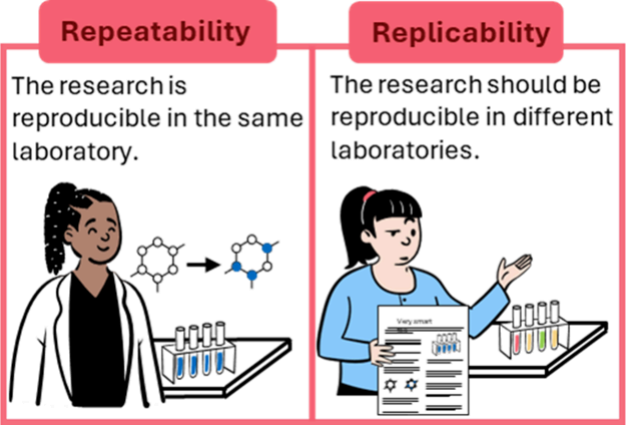
Visual representation of the difference between repeatability and replicability.

#### 2.1.2 Material Characterizations

An extensive characterization through multiple techniques normally follows the synthesis section. The discussion on the material’s characterization invariably embodies a significant portion of the results section. Experimental outcomes are commonly presented in the main body of the manuscript, whereas instrumental details and specific measurement conditions are usually documented in the SI.^21^

Careful and ethical conduct is essential when performing characterizations, as the potential for questionable research practices is always present, and researchers may inadvertently engage in these practices even without explicit intent (Figure 4).^41–43^ For example, “*cherry-picking*” (namely, selective data reporting) is particularly devious. It is not always possible to univocally characterize a powder produced in a (semi)­gram scale, such as CNs, with techniques that analyze just an infinitesimal fraction of the whole sample. At times, the observations done on small regions of the sample are arbitrarily extended to the whole material, while they should be instead combined with other techniques. Possibly, the measurements should be repeated after some sampling schemes from the synthesized lot (for instance, take three small portions of the solid from different spots and repeat the analysis three times). A minimal statistic can provide significant improvement in the correct interpretation of the results (Figure 4).

**Figure 4:**
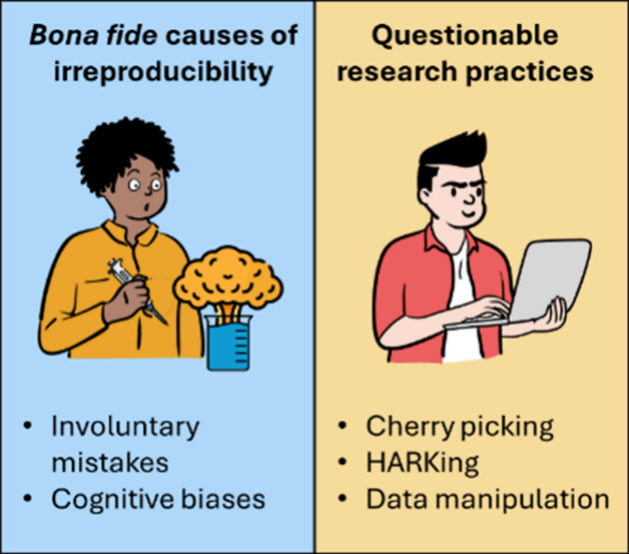
Visual representation of the main causes of irreproducibility in material synthesis.

Another regrettable practice is HARKing, which consists of making hypotheses after the results are known. In other words, changing the hypothesis when data disproves the original one with the sole goal of publishing.^44^ We should be clear on this aspect. Adjusting or revisiting ideas on account of the characterization results is, *per se,* in line with good science, adhering to the “experiment-prove-hypothesis” model. The problems arise when the whole work is based on one incorrect hypothesis and last-minute changes are made so that the data gathered are as descriptive as possible. Such changes are often made to specific parts of the articles and then somehow quickly race over, without considering whether they may invalidate the whole discussion. An even worse scenario takes place when obvious results are forced to fit into the original hypothesis, leveraging on obscure or out-of-context speculations (Figure 4).

In many cases, these situations occur because many laboratories lack the necessary instrumentation to fully characterize materials, so samples are sent to external facilities for analysis. Unfortunately, this is often done just when the synthesized material shows the desired catalytic performances, so the catalytic activity may be ascribed to the wrong hypothesis for most of the time, with predictable consequences. We expand below the discussion in dedicated sections for different types of characterization techniques in relation to reproducibility (Figure 5).

**Figure 5:**
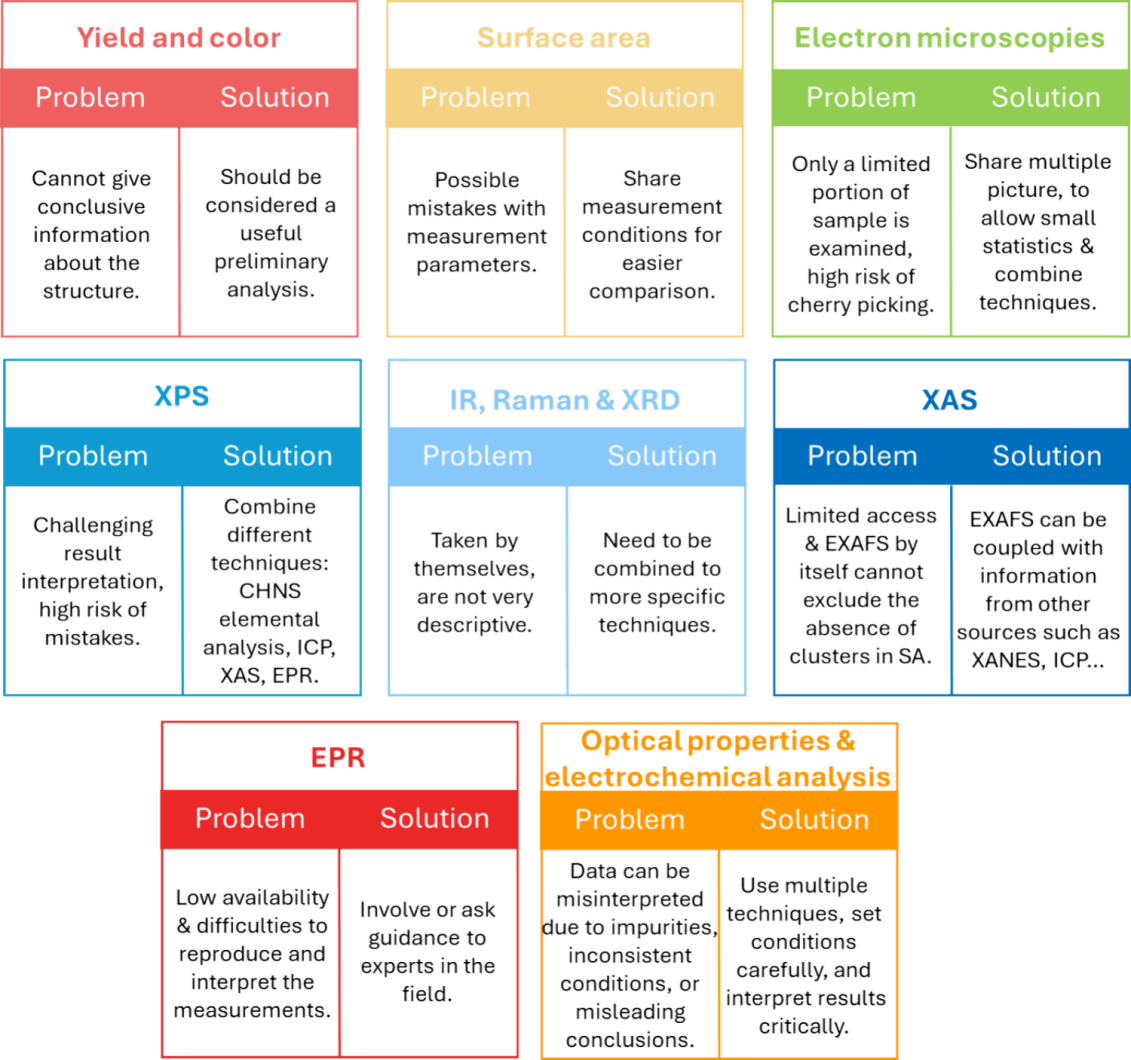
Scheme of the main characterizations reported for carbon nitride. For each technique, the main feature of the measurement that hinders reproducibility and some suggestions to solve the problem are given. (SA: Single Atom.).

##### 2.1.2.1 “Macroscopic” Analysis of CN Materials

The color and the yield of the CN material is worth specifying. This may seem an outdated criterion, and has no analytical value, but the reliance on direct examination of compounds through visual, olfactory, or tactile perceptions is the longest-standing approach in chemistry and can still be of practical help. While visual analysis through reporting the color of the CN material (for instance, adding a photograph of it) cannot be used to understand anything about the structure, it can function as a warning message, should the material behave differently from what expected. For CNs, chromatic shades of the typical yellow color could be linked to a difference in the band gap due to structural alterations. Therefore, the authors may have a feeling about possible unintended alterations of the desired structure by simply inspecting and comparing colors before proceeding to other instrumental techniques. Similarly, significant differences between the yields of two batches of the same material could indicate unintended variation of the synthetic conditions (e.g., differences in temperature in the furnace or in gas flows). In this case, repercussions on the CN’s properties are possible.

##### 2.1.2.2 Surface Area Analysis

Surface area is a fundamental parameter in heterogeneous catalysis, strongly influencing the reaction kinetics. Bulk graphitic carbon nitride typically exhibits a rather low specific surface area, on the order of 10 m^2^ g^–1^ (with melamine and Dicyandiamide, rising to ∼ 70 m^2^ g^–1^ when derived from urea).^45,46^ Such limited surface area directly constrains the number of accessible active sites and thus the overall catalytic efficiency.^47,48^ In many cases, surface area is also an index to confirm the effectiveness of the preparation procedure. For example, mesoporous carbon nitride (mpg-CN) requires a hard template and precise control of the experimental settings to assemble large surface area structure (from 150 to 400 m^2^ g^–1^).^49^ Therefore, large deviations from the typical surface area values may indicate a faulted synthesis, for instance an ineffective removal of the silica template. Similar considerations can be derived for other popular techniques to obtain high surface area carbon nitrides, such as thermal exfoliation.^47^ The values of the surface areas critically depend on the N_2_ physisorption measurement conditions. In particular, the degassing step is very important: a too low degassing temperature may fail to completely remove all adsorbates (particularly water), while exceeding in degassing temperature could alter the material’s textural characteristics, through pore collapse or functional groups removal. Thus, given the high relevance of surface area in CN catalytic applications, we recommend that all measurement conditions (e.g., degas temperature and time, type of probing gas) are diligently included in the paper.

##### 2.1.2.3 Spectroscopic and X-ray Techniques for Initial Screening

Spectroscopic methods such as infrared (IR) and Raman spectroscopies, as well as X-ray diffraction (XRD), are particularly practical for an initial screening of bulk and modified CN material. These techniques are widely available in many laboratories and can give unique, recognizable patterns or “fingerprints” that are very useful for reproducibility: for example, they help verify whether the material matches literature reports (verifying material replicability), and check if the synthesis yields consistent results across different replicates.^50^ However, extracting useful information on the chemical composition or the structure of CNs from such methods is not always straightforward. In IR, the complexity of the material structure and its inhomogeneity cause peaks’ broadening and overlapping, making peak assignment challenging. On the other hand, Raman spectra measured at common laboratory laser wavelengths are affected by significant fluorescence of the material, which makes it difficult to detect the characteristic peaks. Finally, the typically low crystallinity of conventional PCN limits the structural information achievable from XRD. Unless specific techniques with modern advanced instruments are used, we discourage excess of speculative discussion in the characterization with these techniques.

##### 2.1.2.4 Electron Microscopy for Structural Analysis

Textural and morphological information on the CNs can be easily derived from various types of electron microscopies, such as scanning electron microscopy (SEM), transmission electron microscopy (TEM), high-resolution transmission electron microscopy (HR-TEM), scanning transmission electron microscopy (STEM) and atomic force microscopy (AFM). Despite being easy to analyze and informative, these techniques analyze small portions of the sample rather than the bulk and are easily subjected to the above-mentioned “*cherry picking*” practice. Information becomes useful to others if a statistical analysis is performed and presented to provide a robust and more comprehensive vision of the material.

##### 2.1.2.5 XPS and Complementary Techniques for Chemical Analysis

A cross-check with multiple techniques is necessary to disclose the chemical composition and atom arrangement of the CN materials. X-ray photoelectron spectroscopy (XPS) is highly valuable to understand the elements’ chemical environment within the superficial layers of a material (up to a few hundred nanometers, depending on the X-ray source). Thus, information about the relative quantity of C and N atoms and their valence states can be derived by the deconvolution of the signals, drawing hypotheses on the type of structure and functional groups and metal and nonmetal atoms if present. However, it should be remembered that XPS can only probe the outer layers, while being silent on the inner bulk. Second, this technique is liable to mishandling of the data processing stage, since the challenging peak fitting is often at the origins of misinterpretations. Regarding the analysis of the inner layers of CNs, Zhang, Loeffler and colleagues^51^ developed an argon cluster ion etching method in order to gain information on the deeper layers; while very powerful, this method is rather advanced and yet to gain widespread use, so that additional characterization with XPS or other more common methods are desirable to strengthen the conclusions.^52–54^ Quantitative assessments may be complemented by CHNS elemental analysis, which allows the extrapolation of the C/N ratio in the bulk material (as well as S and O). In addition, Inductively Coupled Plasma (ICP) with Mass Spectrometry (MS) or Optical Emission Spectroscopy (OES) detectors can be powerful for determination of metals. In this regard, we recommend checking which are the most suitable protocols to efficiently solubilize the targeted analyte in a quantitative way (e.g., strong acids, *aqua regia*, microwave-based digestions, see further details in Section 2.3.2 *ii*).^55^

##### 2.1.2.6 XAS for CN-Based Single-Atom Catalyst Characterization

XAS is a fundamental modern technique for probing the electronic and local structure, and, in the case of CN, its utility becomes particularly significant for characterizing single-atom-modified carbon nitrides (SA-CN). Single-atom catalysts (SACs) are currently at the frontier of heterogeneous catalysis, but their identification is a challenging task since it eventually requires ruling out any concomitant presence of aggregates of the metal.^56–60^ This aspect is crucial for a correct definition of the active site. XAS, and in particular the X-ray Absorption Fine Structure (EXAFS) portion of the spectrum, provides information about the scattering effects experienced by the photoemitted electrons at varying wavelength. The chemical surroundings of the targeted chemical species are then defined by fitting the EXAFS spectrum with an appropriate (although to some extent arbitrary) model. In parallel, X-ray Absorption Near-Edge structure (XANES) provides additional insights into the oxidation states of the metals. However, EXAFS data analysis is not simple, subjective interpretations and excessive parameter fitting can be misleading, and it can also be ambiguous on the true absence of metal clusters.^61^ It often occurs that the single-atom catalysts turn out to be highly dynamic species as they can migrate and rearrange during the reaction itself, and an analysis of the as-prepared material may differ from the truly active species under operational conditions. Hence, further confirmation of the catalyst’s single-atom nature eventually requires advanced *operando* set-ups.^62^ Thus, circumspection should be taken regarding mechanism speculations mainly founded on EXAFS data. A practical bottleneck with XAS is that atomic resolution data requires synchrotron facilities, making this analysis comparably limited in availability to most researchers. It is imperative, therefore, to couple EXAFS with complementary techniques to confirm SAC structures. For instance, modern high-resolution TEM, equipped with aberration correctors and High-Angle Annular Dark Field detectors (HAADF) and operated in scanning mode, can image the single metal atoms and their dispersion on the CN, while checking the possible presence of small clusters. Fourier Transform Infrared spectroscopy (FTIR), particularly if measured by *in situ* Diffuse Reflectance Infrared Fourier Transform (DRIFT) with probe molecules, and solid-state nuclear magnetic resonance (ss-NMR) can contribute to ascertaining the presence of metal clusters.^63^

##### 2.1.2.7 Electron Paramagnetic Resonance (EPR)

Electronic paramagnetic resonance (EPR) gives information on electronic spin states within the material. While already being affirmed and well-known for the detection of free radicals, it proved to be extremely versatile for the study of materials.^64^ EPR allows detection, quantification and definition of the chemical nature of paramagnetic defects/moieties present on the surface of heterogeneous photocatalysts.^65^ The technique has been used to discern the nature and the fate of the photogenerated electrons in CN at the nanosecond scale, eventually elucidating the possible formation of triplet exciton states as a recombination pathway.^66^ Although we acknowledge that the equipment and the data analysis can be beyond the reach of many laboratories, opportunity to employ EPR to tackle the spin aspects and light induced reactivity of the photocatalyst is of high value. A comprehensive review pointing in this direction has been recently published by Actis et al.^67^

##### 2.1.2.8 Assessment of the Optical Properties and Electrochemical Analysis

For applications in photocatalysis, which is the lion’s share of CN articles, the evaluation of the optical features allows a better understanding of the possible mechanism and the photocatalytic efficiency. As also stressed in one editorial by *ACS Appl. Mater. Interfaces*,^68^ most authors often tend to present the performances using nonquantitative terms, which makes it hard to properly rank the catalyst; therefore, a critical report of the characteristics and properties of the material are needed to increase clarity. Diffuse Reflectance Spectroscopy (DRS) is typically used to determine the bandgap of semiconductors. It is generally combined with XPS, Ultraviolet Photoemission Spectroscopy (UPS) or electrochemical methods (such as Mott–Schottky analysis) to assign the energy of the valence and conduction band edges. It is worth noting that in the absorption spectrum recorded with DRS, the origin of certain bands can be due to foreign species such as organic impurities. Authors are encouraged to carry out a critical inspection of the possible nature of organic impurities, according to the synthetic protocols adopted. For this purpose, a few experimental trials can then be performed by means of UV–vis absorption spectroscopy.

Photoluminescence (PL) is another important technique for probing the optical and electronic properties of CNs, offering direct insight into photocatalytic performance. Analysis of PL spectra allows estimation of the optical bandgap and identification of defect-related recombination centers, thereby probing charge-separation efficiency.^69,70^ However, this technique presents several challenges: the inherently weak PL emission of CNs complicates quantification of defect states and band edges; overlapping, broad emission bands hinder peak deconvolution and thus lead to ambiguous interpretations; and, because PL reports only on radiative recombination events and ignores nonradiative pathways, its ability to assess true photocatalytic charge-separation efficiency is inherently limited.^71–73^

Electrochemical measurements, such as onset potentials or current–potential curves observed during periodic discontinuous (on–off) illumination can provide information on the charge transfer dynamics occurring in the catalyst. In parallel, Mott–Schottky analysis allows to calculate flat band potential, which is useful to understand better the charge transfer dynamics. All these data are of great utility, but our impression is that, in many cases, there is a tendency to abuse the information, in particular for the justification of otherwise doubtful mechanisms. Conditions for charge transfer occurring in photochemistry are not always equivalent to those for electrochemistry. Charge separation in photocatalysts leaves the materials with a near-zero net charge, while the application of an external bias *charges* the catalyst to different degrees depending on the applied potential. Hence, the charge transfer processes occur under different electric fields in the two cases. Moreover, in the case of pure heterogeneous photocatalysis, the CN is dispersed in a liquid medium (which could also be an organic solvent), while the electrochemical setup requires conductive support to immobilize the catalyst and an aqueous electrolyte solution. The different environment may alter the charge carrier transport phenomena of the CN through interfacial effects, with different structures of the electrical double layer and modified diffusion/migration coefficients. For these reasons, we advise making cautious use of electrochemical (or photoelectrochemical) analyses, carefully accounting for the conditions used. Some cross-checks, such as repeating the electrochemical experiments in different electrolytes, depositing the CN of different types of supporting electrodes, checking the pH, and changing the deposition method on the electrode could be very informative for a reliable interpretation of the material’s properties.

Finally, to have a complete look at the efficiency of the catalyst, the exciton lifetimes must be measured. This is done through pump–probe techniques such as Transient Absorption Spectroscopy (TAS), which describes the dynamics of the excited states and of other photophysical processes.^74^ Such a technique provides a complete understanding of charge carriers’ behavior after the light pulse, especially when comparing simple PCN with modified counterparts, and its use is strongly suggested as a complement to the mechanistic discussion.^75,76^ Nonetheless, the analysis of these spectroscopies requires careful consideration: there exists a strong dependence on the sample preparation, as layer inhomogeneities can lead to irreproducible results, but also an excessive presence of defects can make it difficult to model and interpret charge carrier dynamics.

#### 2.1.3 Stability Tests: Spent Catalyst Characterization and *In Situ*/*Operando* Studies

A major advantage of heterogeneous catalysis (over the homogeneous counterpart) is that the catalyst can be easily recovered and recycled after its use, which implies that it should not undergo decomposition during the reaction.^77^ This is an important feature to investigate for a potential transition to industrial-level applications. Thus, recyclability tests and postcatalysis characterizations of the recovered catalysts should always be performed and included in the discussion.

A thoughtful *operando* study is also highly valuable to assess the catalyst’s stability and to disclose key mechanistic steps of the reaction. Advanced characterization techniques based on *in situ* or *operando* modes are indeed becoming more and more popular as they provide a more truthful description of the catalytically active species.^78–80^ Unfortunately, access to these types of facilities is not yet so widespread, and the measurements can also be quite time-consuming. When reported, *in situ* or *operando* analyses must be described in detail, including the type of cell/sample holder and the specific analytical setup, and with no omission of the results contrasting the original hypothesis.

### 2.2 Reaction

In the following section, we will focus on the general parameters that control catalytic performance and require careful analysis. Reproducibility of catalytic performance is a notorious problem in CN-catalyzed photoreactions.^20^ A set of good practices would be of great aid to improve reproducibility.

#### 2.2.1 Control Experiments

Control experiments are the roots of any catalytic investigation and need to be carried out at the very start of any work, taking care that all the control experiments are done under conditions where the maximum yield of the catalyst is within the range of 30–50% for making a meaningful comparison of the changes in activity.

As good practice, all control experiments during the screening and optimization stage of a photocatalytic reaction should be reported, most of the information being provided in the SI. The main text of the manuscript could instead be limited to reporting the control experiments in the optimized conditions. If significant observations are made, like the degradation of the reagent at a certain wavelength or the reactivity quenching with a certain additive, the information should not be ignored, even if no precise conclusions can be drawn about it. In fact, others may find the data useful to confirm other types of hypotheses in other contexts. Another best practice when reporting a new photocatalytic reaction is to test one or more benchmark photocatalysts, preferably commercially available materials, under the same conditions used for CN, so that their activities can be directly compared. In this way it is possible to demonstrate where CN material stands in terms of activity and help readers assess practical relevance of the study.

Below, we present a tentative list of typical control experiments to be performed at any stage of the investigation (Figure 6b):

- reaction in the absence of photocatalyst;
- reaction in the absence of additives;
- reaction in the absence of light, it is preferable to perform the reaction both at room temperature and at an relatively high temperature (for instance: 50 °C) to exclude thermal contributions from the light source;
- reaction under a different atmosphere, e.g., by testing the reaction under an inert gas to see if it was originally performed in the presence of air/oxygen to verify the role of oxygen and vice versa.
- different batches of the same catalyst should be tested, to verify the reproducibility of the catalytic performances (i.e., yield, selectivity, productivity, among others);
- changing sacrificial agent (when applicable) or any other additive (such as acids and bases);
- reaction using a different wavelength (optional), ensuring that the material absorbs in that region, while the reagent does not.

**Figure 6:**
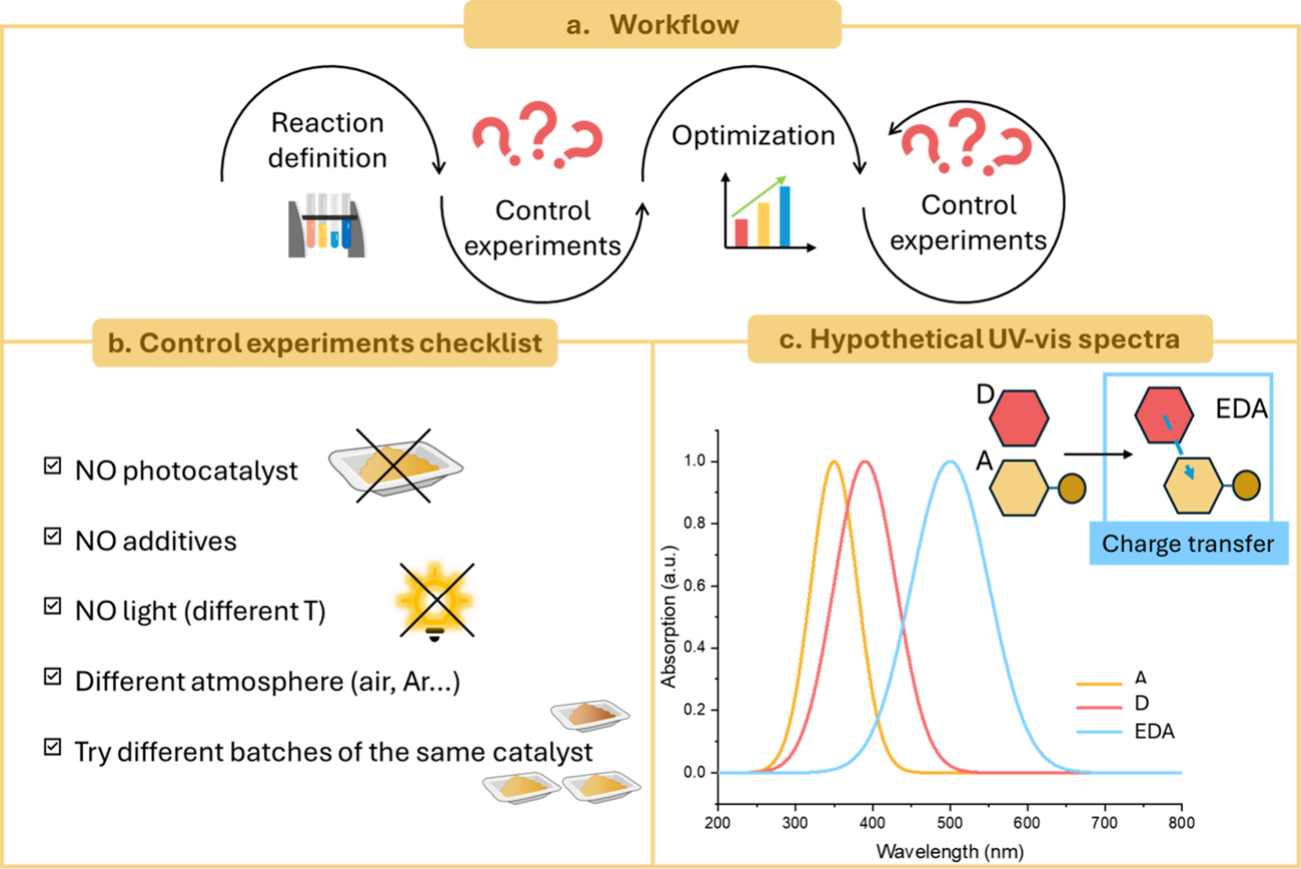
a) Visual representation of workflow during reaction optimization. b) Checklist of the control experiments to carry out while studying the reaction. c) Possible UV–vis spectra of two hypothetical organic molecules, D: donor and A: Acceptor, and their adduct called “electron donor–acceptor (EDA) complex” that absorbs at higher wavelengths, in the visible range.

The control experiments should also be reassessed throughout the condition’s optimization stage, because changes in the solvents or additives can open new reaction pathways. Hence, it must always be verified that under such an optimized reaction regime, the control experiments remain negative with respect to the full catalytic conditions (Figure 6a).

As example, we illustrate the potential pitfalls associated with changes in reaction conditions by examining the use of various brominating agents. *N*-bromosuccinimide (NBS), KBr and HBr are all common brominating agents in photocatalysis. However, when NBS is used as the brominating agent for aryl compounds, the bromination reaction of aromatic supports may proceed with no catalyst’s assistance. This is due to the fact that HBr traces, usually present in NBS, cause the formation of molecular bromine (from NBS itself) that, under light irradiation, undergoes homolytic cleavage. The as-formed Br· radicals can then cause a free-radical halogenation reaction.^81,82^ In contrast, KBr and HBr generally require a photocatalyst to proceed with the reaction. Since KBr and HBr are weaker brominating agents, the bromination can be obtained only on highly activated aromatic rings bearing strong electron-donating groups. Because of this significant difference in their application, comparing photocatalytic bromination with different bromination agents, such as NBS with KBr or HBr, could mask the role of the catalyst and lead to a wrong conclusion.

Similarly, when a hole scavenger is used to facilitate photoreduction reactions, its contribution to the reaction should be carefully recognized and discussed. Common sacrificial donors are sulfides/polysulfides, ethylenediaminetetraacetic acid (EDTA), triethylamine, and alcohols. However, these molecules can play multiple roles in the reaction. For example, triethylamine can alter the pH of the medium, thereby introducing an additional variable into the experiment, while EDTA, being a powerful complexing agent, may cause unexpected side effects if metal cations are involved. Control experiments, performed after the screening of the various species, could help to avoid such pitfalls.^83,84^

As good practice to increase reproducibility, a complete description of the reaction setup is demanded. First, the reactor is very important: size, kind (e.g., Schlenk tube, sealed tube) and material (such as borosilicate glass or quartz) must be specified because they affect the optical path as well as the light penetration, thus changing the quantum yield and the photocatalytic efficiency.^85,86^ For similar reasons, it is also of pivotal importance to specify and to keep constant the geometrical distance between the light source and the reactor.^86^ It is imperative that the light intensity remains constant throughout the investigations. Full technical details of the light source should be provided, attaching in the SI a spectrum of the emitted wavelengths (if possible), and optical surface power density (expressed in mW cm^–2^), especially for quasi-monochromatic LEDs or lamps (e.g.; Kessil lamps). Additionally, the heat generated by the radiant energy must be efficiently dissipated out of the reacting system using fans or air conditioning. This is to avoid any contributions from thermal energy (if evaluation of pure photocatalytic activity is the objective), therefore measurement of the temperature during the reaction needs to be monitored.^86^

Although often ignored, the stirring rate is an important parameter to ensure homogeneous catalyst distribution, but the choice of the stirring bar is even more crucial. In fact, “*stirring bar catalysis*” is a known pitfall, related to the tendency of polymer-coated laboratory items to adsorb metals. Ideally, the use of brand-new stirring bars is advised, if possible, when a completely new reaction is explored. If this practice becomes too unsustainable (e.g., the number of reactions is too large), at least the repetition of the reaction with different stirring bars is recommended.^55,87^

Another generally overlooked task is to measure the UV–vis spectra of the reagents, as dissolved in the same solvent as the optimized conditions, as well as those of the complete reaction mixture (filtering insoluble components). In fact, these tests serve to verify that none of the substrates or intermediates absorbs at the wavelength range emitted by the light source, so that a possible direct activation of the substrate by light (without catalyst) is ruled out. Furthermore, the association of an electron-rich substrate (named “donor”, D in Figure 6c) with an electron-accepting molecule (“acceptor”, A in Figure 6c), which can generate a molecular aggregate called “electron donor–acceptor (EDA) complex”, need always to be checked. Compared to the precursor molecules as individuals, this complex typically shows a red-shifted absorption peak that often falls visible light (Figure 6c).^88^

Upon light absorption, D may provide an electron to A, leading to the formation of a radical-ion pair, which can initiate organic transformations in solution even in the absence of a catalyst. UV–vis spectra of the mixture of reagents can help to recognize this problem.^88,89^

Finally, there are two important pieces of information related to the reaction conditions that should be included in the main text of the article. The first refers to the number of times that the reaction was repeated: we believe that a triplicate is a reasonable compromise to obtain a reliable standard deviation for each reaction without exceeding with experimental load in the work. Second, it is relevant to indicate whether the yield of the reaction was averaged over different catalyst batches.

#### 2.2.2 Reaction Conditions and Optimization

The reaction optimization is a crucial stage of catalyst development, and it could be rather complex. Many parameters can be adjusted according to a logical scheme guided by a step-by-step processing of the results of these deviations. A list of possible parameters that can be tuned to enhance the reaction performance includes (Figure 7a):

**Figure 7:**
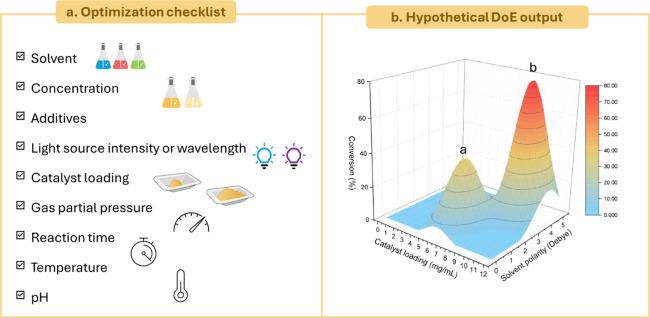
a) Checklist of the experiments to carry out while optimizing the reaction. b*)* Hypothetical DoE output for a generic reaction (varying solvent polarity and catalyst loading as variables) in which there exists a relative (a in the graph) and an absolute maximum (b in the graph).

- change of solvent;
- change of concentration;
- change of reaction stoichiometry;
- variation of type of additive and/or of their concentration;
- adjustment of light source (intensity or wavelength);
- change of catalyst loadings;
- change in gas partial pressure, if a reagent is present in this form;
- extending or reducing the time for the reaction;
- change of reaction temperature;
- change pH of the reaction environment.

The most used approach for reaction optimization is called One Factor at a Time (OFAT), in which each factor is iteratively optimized while keeping the others fixed. Despite its conceptual simplicity, this methodology is laborious and time-consuming, and the results are strongly dependent on the initial conditions. Indeed, this process can lead to a local optimum (for example, the peak “a” in Figure 7b), while the “real” optimal conditions (corresponding to the absolute best results, which is “b” in Figure 7b) could be missed. An alternative to the OFAT approach is the “Design of Experiments” (DoE). This is a statistical approach that generates a mathematical model that links the experimental results to the reaction parameters. Initially, the model proposes a pool of preliminary experiments, in which multiple factors (both quantitative and qualitative) are varied simultaneously. Eventually, a series of new experiments can be integrated to achieve the best performance. The simultaneous screening of the variables enables the discovery of interactions with each other and allows one to assign the optimal reaction conditions (Figure 7b) with a reduced total number of experiments as compared to the OFAT way. However, this method is complex, has many mathematical drawbacks, and the number of required experiments increases nonlinearly with the number of variables, so it has yet to achieve widespread popularity.^90^

Finally, the scalability of the reaction can enhance reproducibility, as it provides a means to *corroborate results* under conditions that more closely resemble practical applications. Performing the reaction at the gram scale (typically 1–5 mmol, depending mostly on the substrate’s molecular weight) adds value to the article, given the known limitations of scaling up photocatalytic methods. Clearly, results obtained on a small scale cannot be assumed to translate directly to larger scales, since many factors hinder reproducibility and many of these do not scale linearly with reaction size. For example, the change in the dimension of the reactor and the increased volume of mixture cause difficulties in reproducing the light distribution, mass transfer and temperature gradient across the reactor.^91,92^

#### 2.2.3 Scope of the Model Reaction

In some senses, the scope of the model reaction represents a form of *corroboration* of results. However, if mechanistic details have not been thoroughly explored, it is challenging to explain the activity trends through substrate variation. To this end, control experiments are essential checks that could straightforwardly help in avoiding wrong hypotheses. The oxidation of xanthene and fluorene, respectively, to xanthone and fluorenone is an interesting case study. These two compounds are often found in the scope of photocatalytic reactions with CNs, even though it is known that the photochemical oxidation of these molecules under certain reaction conditions can proceed without catalyst.^93–98^ On the other hand, there is generally reluctance in reporting the experiments that did not prove successful or in full line with the main hypothesis. This is a direct consequence of “publication bias” described before in text: authors avoid publishing the negative results, to increase the chances to publish. However, transparently reporting substrates that fail to react not only enhance the rigor of the work but also could contribute to the scientific importance of a paper, as failed experiments may inspire refined hypotheses on the mechanism and trigger new research on the topic, leading to scope expansion or improved activity. All things considered, we feel it is necessary to rethink the importance of the reaction scope in scientific papers, exploiting those results in a much broader way.

Finally, articles should contain sufficient details of the reaction conditions of each different substrate, which are part of scope exploration. Usually, this is all reported in the SI (see Section 2.3.3), so it will be treated later in this manuscript. However, the most important information is, for example, the name, quantity, purification conditions, as well as the characterization data to confirm the identity and purity of the products and the identity of the possible byproducts.

#### 2.2.4 Photocatalytic Reaction Mechanism

The validation of the mechanism is a stage that needs to be taken very seriously based on multiple experimental pieces of evidence and avoid taking previously reported hypotheses for granted.

In heterogeneous photocatalytic reactions, the initial step involves the activation of the photocatalyst with light. Upon absorbing photons with energy equal to or greater than its bandgap, the photocatalyst reaches an excited state in which excitons (electron–hole pairs) are generated. These charge carriers are then physically separated and subsequently utilized to drive redox reactions.^14^ Possibly, the presence of secondary catalysts (sometimes defined as cocatalysts), can improve catalytic rates, although the exact role of the cocatalyst must be carefully studied. As a general view, the cocatalysts serve to mediate electron transfers, to facilitate charge carrier separation, or to chemically promote the reaction by adjusting substrate adsorption dynamics. They may also enhance reaction rates or selectivity by providing alternative reaction pathways or stabilizing reaction intermediates.^35^

After this initiation step, the mechanisms can proceed through diverse pathways, but the very first step must be clearly understood. Common routes in reactions catalyzed by CNs are based on single-electron transfer (SET), proton-coupled electron transfer (PCET), or energy transfer.^99,100^

A variety of experiments can be conducted to determine which of the possible pathways are actually occurring. Once a hypothesis regarding the reaction mechanism is formulated, a comprehensive set of techniques is necessary to support and validate it. A relatively simple check can be done by means of suitable scavengers which target specific species that are proposed to be part of the mechanism, such as photoelectrons, photoinduced holes, singlet oxygen, peroxo- and other types of radicals, and observe if any decrease (or complete loss) of activity occurs. EPR, electrochemical studies, or spectroscopy methods (like XPS, IR and XAS) used in the *in situ* and *operando* configurations are very precious to ascertain or rule out the implication of radical species and to define the exact type of such radicals.^66,101–106^ Moreover, as suggested above (in Section 2.1.2), TAS can be used to investigate processes such as electron transfer and energy transfer since it tracks the electrons’ pathways and rates at which electrons move within the material or toward adsorbates.^107,108^

In addition to experimental validation, theoretical calculations can support reaction mechanisms by predicting energy changes associated with each reaction step.^109,110^ Whether the predicted energy changes align with the observed values, the proposed mechanism results are bolstered and receive further proof. When these calculations are present, the model and the chosen variables need to be fully described in the SI.^111^ However, computational analysis is beyond our purpose and will not be further discussed, so we recommend checking the literature already available on this topic.^112–116^

Evaluation and verification of reaction mechanisms should not be discouraged by the presence of documented formulations in the literature with other catalysts. This careful scrutiny ensures that the unique characteristics of your specific catalyst are considered, potentially revealing new and valuable reaction pathways. However, it should be taken into account that the complexity of the system usually hinders a complete understanding of the mechanism, and future discoveries may confute or enrich it. As a matter of fact, it happens sometimes that literature offers multiple interpretations of a mechanism: for example, in the air-mediated photooxidation of sulfides to sulfoxides with carbon nitrides, authors disagree on a single pathway, often due to the varying performance of the different catalysts presented. In such cases, check tests using scavengers and control reactions are required to identify the most reliable mechanism.^14^ Another common difficulty is the presence of competitive pathways, such as overoxidation (which is common in alcohol oxidations). If any of these side reactions is suspected, careful studies should be done in order to recognize and describe it, eventually including a complementary discussion on the strategies required to hamper these secondary pathways.^117,118^ Possibility of detecting potential gaseous products by gas-chromatography should also be considered in many cases.

### 2.3 Supporting Information (SI)

As mentioned above, the importance of SI should not be underestimated, as it is the primary resource for gaining more experimental insights into the article. Since the main text of a scientific article must be self-contained due to length restrictions or to improve readability, it is not always feasible to include all the detailed information necessary for a comprehensive description of the work and, most importantly, to facilitate its reproducibility. Therefore, the SI serves as a repository for materials such as detailed experimental procedures, extensive data sets, additional figures and tables, and multimedia content that do not fit within the main text. This approach enables readers to replicate the study and explore specific aspects in greater depth.

The additional space provided by the SI is significant in multidisciplinary research. While integrating diverse disciplines is essential for advancing research, it often results in complex studies that require detailed explanations that pose challenges for effective communication and comprehensive reporting. This aspect becomes especially relevant when journals specialize in a particular field, often applying less stringent standards for reporting data and experimental details from disciplines outside their primary focus. This imbalance can limit the information provided, potentially compromising reproducibility. The SI offers a valuable space for authors to elaborate on those underrepresented aspects, ensuring that readers interested in them have access to comprehensive and detailed information.

#### 2.3.1 How to Structure the SI

This section focuses primarily on the “printable” SI, which typically includes the supporting tables, figures, methods, equations, notes and (in some cases) additional discussion (Figure 8). All that concerns multimedia files and other resources should be prepared and attached at the discretion of the journal’s guidelines, as we will mention in Section 2.3.5.

**Figure 8:**
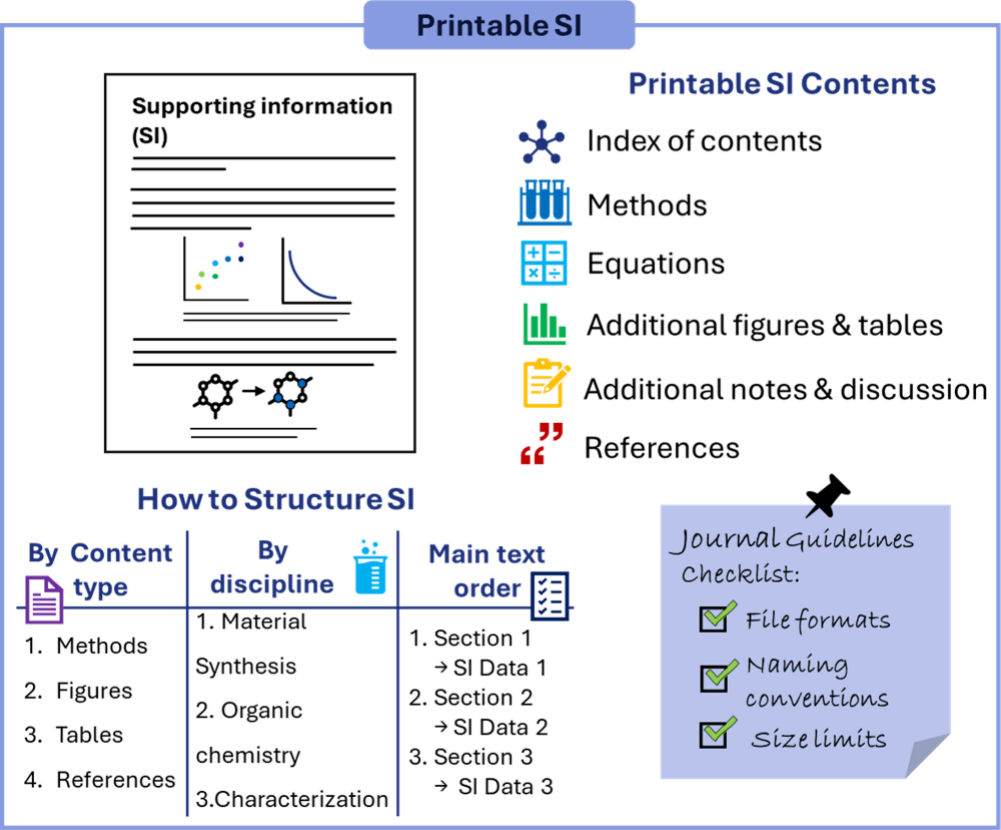
Schematic overview of how to prepare the main file of the SI. The diagram outlines the typical contents to be included, presents alternative strategies for structuring the SI, and provides a checklist of key requirements based on journal guidelines.

While the SI is sometimes reduced to a series of figures with brief captions, it holds the potential to serve a more meaningful and comprehensive role in the scientific narrative. Extensive descriptive text or conventional manuscript sections (such as Introduction or Discussion) are generally inappropriate. Nonetheless, a precise structure of the additional data, with an index of the content, could be added at the beginning to facilitate quick access to specific information.

In general, the SI should mirror the flow of the main text to maintain coherence, especially when it includes references to specific figures or data points in the article. At the same time, a well-organized SI allows readers to locate essential details without having to constantly consult the main manuscript. This is particularly useful for collateral aspects of the research that, while not central enough to be fully discussed in the main text, are crucial for the completeness and reproducibility of the study (Figure 8).

There are several possible strategies for organizing SI. One option is to separate the material synthesis and characterization from the organic chemistry section, which includes reactions and their corresponding characterization data. This format is especially suitable for multidisciplinary studies, where the SI often includes extensive synthetic procedures, yields, molecular weights, and NMR spectra, which are all essential for validating the results. Alternatively, the SI may be divided by content type, grouping methods and instrumental procedures separately from figures and tables. Another effective approach is to present the characterizations and procedures in the same order in which they appear in the main text, associating each paragraph with the relevant figures and/or tables for ease of reference.

As a preliminary step in preparing the SI, it is convenient to consult the author guidelines provided on the journal’s Web site, where details regarding file naming conventions, acceptable formats, file size limits, as well as the content and purpose of the SI are specified (Figure 8). In most cases, the journal’s guidelines also provide instructions on how to format references within the SI. When referencing previously published work (e.g., syntheses, reactions, or characterization results), proper citations should be included regardless of whether the journal provides explicit directives. It is important to note that references within SI are not hyperlinked to the main publication and do not contribute to citation metrics; therefore, their inclusion should be considered thoughtfully and used to support transparency rather than citation performance.

#### 2.3.2 Contents of the SI

Once the structure of the SI is chosen, the next step is to create the actual content. While some ideas have already been referenced multiple times throughout the manuscript, this section aims to provide a comprehensive overview. Please note that some of these elements will be discussed in greater detail in the following sections.

##### i Description of the Experimental Procedures

All materials and synthesized compounds that are not commercially available must be accompanied by their detailed experimental protocol. This should include clear, step-by-step instructions specifying all reagents, solvents, and materials used, along with their quantities, purities, and sources. To ensure full reproducibility, it is essential to report the suppliers and specifications of all purchased reagents and key consumables, such as TLC plates, chromatography columns, and filtration materials.

Any specific conditions (e.g., temperature, pressure, atmosphere) and techniques employed should be clearly stated, including information about the glassware used, reaction times, purification steps (e.g., washing procedures), and yields. Where applicable, schematic representations of the synthetic routes should also be included. Finally, appropriate literature references need to be cited, in accordance with the specific guidelines of the journal, if such methodologies were previously reported.

##### ii Descriptions of Experimental Setups

Photographs of custom-built setups, sample arrangements, or intermediate stages of synthesis can be highly beneficial. In addition, as mentioned earlier in the text, detailed textual descriptions of all relevant equipment (e.g., crucibles, furnaces, and reactors) should be provided regardless of whether images are included, to ensure that the procedures can be accurately replicated.

##### iii Sample Preparation and Instrumental Parameters for Characterization

Sample preparation is a critical yet often underreported aspect of experimental methodology, with significant implications for the reliability and reproducibility of analytical results. Each characterization technique has specific requirements and sensitivities, and the preparation protocols must be tailored accordingly.

For instance, the preparation of grids for TEM imaging involves several variables, including the grid material (e.g., copper, nickel, or carbon), the type of coating (e.g., continuous, porous or lacey carbon), as well as the solvent and concentration of the dispersion used to deposit the sample. These parameters can influence the material’s distribution on the grid image, affecting quality and data interpretation, and should therefore be reported in detail.

Another illustrative example is the preparation of samples for ICP analysis, especially when quantifying metals in carbon-based materials. This process may require pretreatments at high temperatures (e.g., 900 °C) to remove the carbon matrix, followed by acid digestion using *aqua regia*, nitric acid, hydrochloric acid or hydrofluoric acid. The resulting solution must be appropriately diluted with ultrapure water and filtered to obtain a clear, measurable sample. It is also essential to include acid blanks, as trace metal contamination in acids can affect the accuracy of the measurements.

Similar attention should be given to other techniques. For physisorption measurements, degassing conditions must be specified (as explained in Section 2.1.2.2), as well as it should be clear which probe gas and fitting model (BET or Langmuir) have been used to measure the surface area. While using the Attenuated Total Reflectance (ATR) module for FTIR spectroscopy, the type of crystal (e.g., diamond, germanium) used can influence spectral features. Raman spectroscopy requires details such as laser wavelength, number of scans, and acquisition parameters. For XRD, the sample holder type and the software or program used for data acquisition and analysis should be reported.

These details are particularly valuable for researchers who are new to a given technique or material system. Often, first-time users rely on expert guidance, which may not always be fully applicable to their specific materials. Providing comprehensive and material-specific preparation protocols in SI can significantly aid reproducibility and serve as a practical reference for the broader scientific community.

##### iv Supplementary Figures

Figures that are essential for a comprehensive and thorough description of the study, but are not central to the discussion, should be provided in the SI. These typically include UV–vis, IR, and Raman spectra as well as additional high-resolution imaging data such as TEM, AFM, and SEM.

It is important that all figures be original and not duplicates of those presented in the main manuscript. Each should be accompanied by a concise and informative caption that includes a brief interpretation or contextualization of the data, particularly when limited space in the main text prevented detailed discussion.

Where appropriate, multiple images from the same technique (e.g., several TEM or AFM micrographs) should be provided to offer a statistically robust depiction of the material’s morphology and structure. Additionally, as noted earlier, images of spent catalysts (highlighting changes such as morphological degradation or single-atom aggregation) are important for supporting discussions on stability and performance.

##### v Supplementary Tables

Tables should be used to report detailed comparisons, experimental outcomes, and control results that are too extensive for inclusion in the main manuscript. A key application of supplementary tables is the comparison of the performance of the newly developed catalyst with those already reported in the literature, particularly catalysts of similar nature (e.g., other carbon nitride-based systems as well as some relevant homogeneous or heterogeneous catalysts). These tables should highlight relevant performance metrics such as activity, selectivity, stability, and reaction conditions, enabling a clear assessment of the catalyst’s relative advantages. Very often the performance metrics are divergent in terms of the reported parameters, as authors always look for the best-selling point of their manuscript. It is the responsibility of the new work’s authors to study and translate the previously published data into a harmonized set of metrics, in order to have a fair comparison.

In addition, (as mentioned above) tables documenting failed or nonoptimized experiments are encouraged, as they provide valuable insight into the experimental process and help contextualize the final results. These entries should include a brief description of the tested conditions and the observed outcomes. If not already reported in the main text, complete data sets from control experiments should also be included, detailing variations in reaction parameters, the absence of specific components, or the use of alternative materials. All tables should be clearly labeled, include appropriate units, and be referenced in the main text.

##### vi Methods for Product Identification and Isolation

Please refer to Section 2.3.3.

##### vii Quantification Metrics

Please refer to Section 2.3.4.

##### viii Additional Computational Studies

As stated above (Section 2.2.4), the discussion of computational studies goes beyond the scope of this work and will not be further addressed here.

#### 2.3.3 Product(s) Identification and Purification

For each product, the yield and selectivity are the first parameters to report, but also the equations used to estimate them should be specified. Figure 9 summarizes the main techniques used for the identification and purification of organic products, which will be further discussed in this section. The simplest way to calculate the yield starts from weighing the mass of the isolated pure product. Though it may seem simple, it is worth noting that the purity of the product needs to be ensured to avoid the overestimation of activity. First and foremost, the product should be thoroughly dried to remove excess solvents that can remain entrapped, and the chemical structure and purity of the final compound should be confirmed with techniques like Nuclear Magnetic Resonance (^1^H-, ^13^C-, heteronuclear- and ^13^C­{^1^H} bidimensional- NMR) spectroscopy, chromatographic methods (High Performances Liquid Chromatography - HPLC, Gas Chromatography - GC, *etc*.) and High-Resolution Mass Spectrometry (HRMS). In the specific case of NMR, the peaks’ listing, the solvent used, and the spectrometer specifications should always be provided. It is also necessary to include a clear picture of the spectra and the raw data.

**Figure 9:**
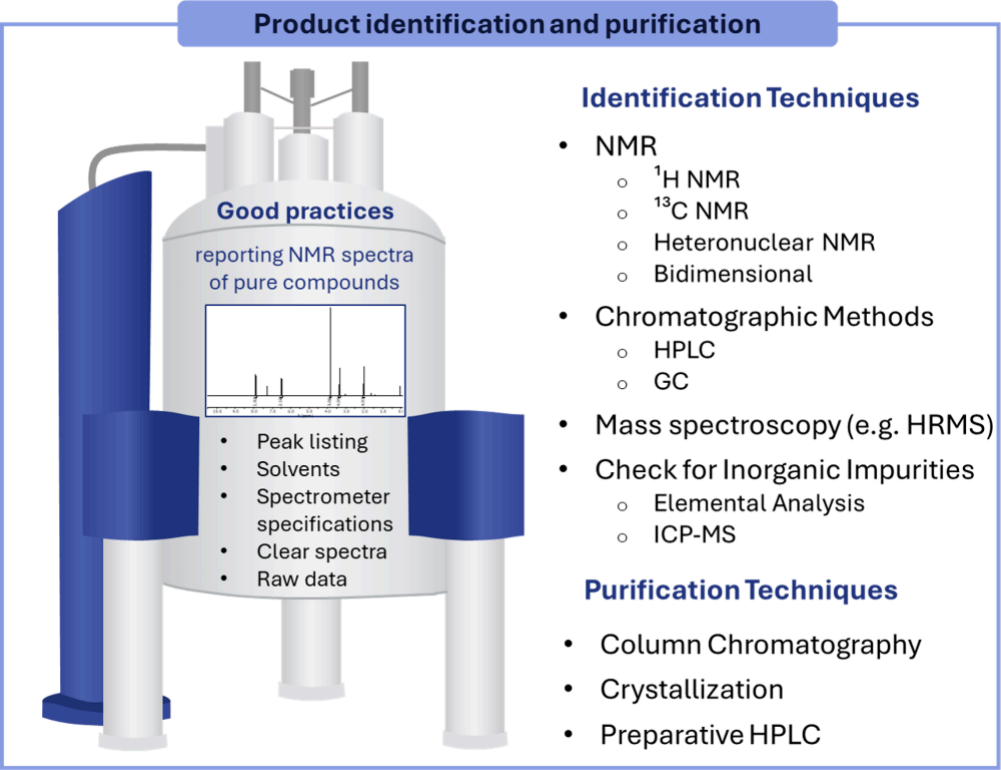
Scheme of the main techniques used for organic products identification and purification, with some good practices related to the reporting of NMR spectra of pure compounds.

Ultimate confirmation of the absence of inorganic impurities (which, for instance, cannot be detected with ^1^H NMR, ^13^C NMR) should be attempted by cross-check characterization such as elemental analysis, ICP-MS or other direct or indirect techniques.^55^

Moreover, during the optimization step, the estimation of the yield through NMR by the addition of an internal standard (IS) can represent a faster and simpler way to derive the reaction yield compared to the “macroscopic analysis” based on mass weighing. The use of an IS is also useful to reduce human error during the purification steps. Some precautions are necessary when choosing the IS: it would be convenient if it had similar physicochemical behavior (e.g., polarity, boiling point) with the analytes, it should dissolve in the deuterated solvent of choice and, most importantly, its NMR peaks should fall far from those of the products and the reagents, to avoid overlapping. In this regard, 1,3,5-trimethoxybenzene and trichloroethylene are classic examples of ISs. The NMR yield using an IS (*Y*_*IS*_ *%*) is calculated as shown in eq 1. In this equation *A*_*P*_ and *A*_*IS*_ are the integrated areas of a selected signal from the product and the IS, respectively. *mol*_*R*_ and *mol*_*IS*_ are the initial moles of the reagent and the moles of IS, while *nH*_*P*_ and *nH*_*IS*_ are the number of protons corresponding to the chosen signal. To simplify the calculation, the term $\frac{{mol}_{IS}}{\frac{A_{IS}\times {mol}_{R}}{{nH}_{IS}}}$ can be set equal to 1, this can be achieved, for example, by using equal moles of reagent and internal standard (*mol*_*R*_ *= mol*_*IS*_) and by arbitrarily setting the area of the IS peak in the NMR processing software equal to the number of protons it represents. Under these conditions, the yield can be directly calculated by dividing the area of the product peak by the number of protons it represents.

$$Y_{IS}\left(\%\right)=\frac{\frac{A_{P}\times {mol}_{IS}}{{nH}_{P}}}{\frac{A_{IS}\times {mol}_{R}}{{nH}_{IS}}}=\frac{A_{P}}{{nH}_{P}}\;\mathrm{when}\;\frac{{mol}_{IS}}{\frac{A_{IS}\times {mol}_{R}}{{nH}_{IS}}}=1$$ 1

In the case NMR is the sole technique used to calculate yield (in the circumstances where isolating the product is not technically achievable within the experimental conditions used), authors should clearly specify this. However, we recommend relying on isolated yield to gain a full picture of the result of the whole protocol.

The method used for the purification should also be described in detail. For instance, the solid phase and the eluent ratio must be reported if column chromatography is used, while a detailed protocol which describes temperatures and solvents should be indicated in case the product is purified by crystallization.

In the specific case of product characterization, data processing can be easily exposed to inappropriate manipulation, and this is a significant concern in scientific research. Evidence suggests that this problem is present and persistent. For instance, the editorial staff of the journal *Organic Letters* has reported that 2–3% of submitted manuscripts show signs of manual peak removal in NMR spectra.^20^

#### 2.3.4 Quantification Metrics

An important part of the SI should be dedicated to reporting the relevant equations. Yield (Y%, eq 2), conversion (C%, eq 3) and selectivity (S%, eqs 4a and 4b) are usually reported as percentages (moles or mass yield of the products is also reported in the SI). Conversion is defined as the fraction of the reagent that has been transformed into the products. This number should be very close to the total yield (i.e., all products observed), to rule out any byproduct or reagent loss. Selectivity is defined (depending on the specific type of catalytic process) as a) the ratio of the amount of a target product to the sum of amounts of all the byproducts, b) the ratio between the product and the reagent or c) the ratio between the yield and conversion.

$$Y\left(\%\right)=\frac{Amount\;of\;product\left(mol\right)}{Theoretical\;amount\;of\;product\left(mol\right)}\times 100$$ 2

$$C\left(\%\right)=\frac{Amount\;of\;reactant\;consumed\left(mol\right)}{Initial\;amount\;of\;reactant\left(mol\right)}\times 100$$ 3

$$S_{a}\left(\%\right)=\frac{Amount\;of\;product\left(mol\right)}{Sum\;of\;all\;products\left(mol\right)}\times 100$$ 4a

$$S_{c}\left(\%\right)=\frac{Yield}{Conversion}\times 100$$ 4b

As stated in previous sections, the energy and intensity of the light employed for the photocatalytic tests must be measured according to standardized methods and specified to ensure reproducibility and enable the calculation of quantum yield (eq 5) or quantum efficiency (QE, eq 6).^119^ Several reviews and perspective articles have suggested harmonized guidelines for reporting activity in heterogeneous photocatalysis.^85^ To provide general context in this regard, quantum yield (QY or Φ­(λ)) is defined as number of defined events, occurring per photon absorbed by the system at a specified wavelength.

Unfortunately, in heterogeneous photocatalysis, the number of absorbed photons is experimentally difficult to estimate not only due to reflection and scattering phenomena but also because the number of active sites of the photocatalyst is not easy to determine experimentally.^120^ For this reason, apparent quantum yield (AQY) is generally preferable, since this is the number of defined events occurring per photon absorbed by the system at a specified wavelength (eq 7).

$$QY\;or\;\Phi (\lambda )=\frac{Amount\;of\;reactant\;consumed}{Amount\;of\;photon\;absorbed}$$ 5

$$QE=\frac{Rate\;of\;reactant\;consumed}{Total\;absorbed\;photon\;flux}$$ 6

$$AQY\;(\%)=\frac{Number\;of\;reacted\;e^{‐}}{Number\;of\;incident\;photon}\times 100$$ 7

A second problem is that to properly determine the QY it is necessary to use monochromatic light. However, the common light sources (both LEDs and Kessil lamps) are usually polychromatic with a certain width (in the range of a few nanometers) of the emitted wavelengths. Therefore, measurement of both the QY and AQY should be considered an estimation.

#### 2.3.5 Multimedia Files and Data Sets

In addition to the “printable” material described so far, SI include multimedia files and data sets that enhance the understanding and reproducibility of the study. Multimedia content, such as videos demonstrating experimental procedures, animations of theoretical models and 3D models, or time-lapse recordings of reactions, can provide valuable visual context that complements the written descriptions. Data sets should be provided in accessible formats, following the specific guidelines of each journal, if present (e.g., CSV, XLSX, TXT) and should include clear labeling, units, and metadata to facilitate reuse and verification. A brief description of the data structure, collection methods, and any preprocessing steps should accompany each data set to ensure clarity and reproducibility.

In addition to processed data, the sharing of raw data files is increasingly encouraged (or even demanded), particularly for characterization techniques such as spectroscopy, microscopy, and diffraction. Raw data allow for independent verification of results and help prevent data manipulation or misinterpretation. However, sharing raw data can be challenging due to the prevalence of proprietary file formats generated by instrument-specific software. These formats may become inaccessible if the associated software licenses expire, posing a risk to long-term data availability.^121,122^ Despite these limitations, depositing raw data in open-access repositories is strongly recommended. Doing so not only supports scientific transparency but also aligns with the growing number of funding agencies that require applicants to outline open access and data management plans as part of their research proposals.

## 3 Conclusions

It has been more than a decade since the idea of slow science emerged, encapsulated in the manifesto that can be summarized as “Science needs time to think. Science needs time to read, and time to fail”.^123^ However, it is clear that this concept does not fit well in modern research practices. The provocative question posed by Prof. Jean-François Lutz in a 2012 Nature Chemistry article remains: “Is the high number of publications merely boosting scientists’ egos, or does it benefit global human knowledge?”.^2^

The present Perspective aims at raising awareness on the necessity to revisit the writing of scientific articles, in order to find harmonized structures and ensure robustness and reproducibility of the results. We believe that stress for publication, dictated by the frenzy of publication metrics, is becoming a serious problem for scientific advancements, and the philosophy of article publishing should look at “*less quantity high quality*” creed. Methods to ensure high quality and utility of scientific reporting on the theme of photochemical organic transformations with carbon nitrides are outlined here as a case study, but the same concepts apply to any experimental work. Here we attempt to provide a general route to achieve the production of a sound scientific article. Suggestions on how to write papers on this topic are provided, and good practices that should improve the reproducibility of the research are herein recommended. Central issues that are mainly responsible for irreproducibility of results are discussed; for example, the main drawbacks related to the most common characterization techniques and the issues related to the reaction scope are investigated. Finally, some biases and bad habits that can interfere with scientific reporting and hinder reproducibility are also addressed throughout the paper. Therefore, the risks of involuntary mistakes, underestimated problems, and questionable research practices are exposed and explained.

A final consideration relates to the fact that someone would argue that Science is still progressing, even at this fast pace, and despite the large number of redundant or incorrect published articles. However, it is crucial to evaluate at what cost the progress occurs within the current paradigm. Beyond the general decline in quality and the increased risk of fraud, several studies report that there exists a prevalence of psychological problems among doctoral students, who are at the forefront of research, and therefore the first to suffer from the publish or perish culture.^124–128^ It is our opinion that addressing these issues is essential for fostering a healthier and more sustainable scientific community.
